# A Course of Lectures on Dental Physiology and Surgery, Delivered at the Middlesex Hospital School

**Published:** 1847-10

**Authors:** John Tomes

**Affiliations:** Surgeon Dentist to the Hospital.


### ARTICLE V.

*A Course of Lectures on Dental Physiology and Surgery, delivered at the Middlesex Hospital School.* By JOHN TOMES, ESQ. Surgeon Dentist to the Hospital.

**(Continued from Vol. vii. No. 2.)**

### LECTURE VII.

ABNORMAL CONDITIONS OF THE FIRST DENTITION—DISEASES OF THE GUMS BEFORE AND DURING THE ERUPTION OF THE MILK TEETH (INFLAMMATION AND INDURATION)—DISEASES OF THE TISSUES OF THE MILK TEETH (CARIES AND NECROSIS)—DISEASES OF THE GUMS AFTER THE ERUPTION OF THE MILK TEETH—DISEASE OF THE ALVEOLAR PERIOSTEUM OF THE MILK TEETH.

ABNORMAL CONDITIONS OF THE SECOND DENTITION—IRREGULARITY IN THE TIME OF ERUPTION OF THE PERMANENT TEETH.

GENTLEMEN:—Since I have directed my attention to the diseases of the teeth, it has been my habit to make notes of the most instructive cases that have come under my notice, and also of those that have been related by authors. In order to accomplish the purpose I have had in view—namely, to gain useful illustrations of the various dental maladies—I arranged the subject into divisions and subdivisions. This classification, which I adopted as convenient for my private use, I shall follow in describing to you the diseases of the teeth. I have therefore prepared a table, the perusal of which will give you the order in which each malady will be discussed.

In accordance with this plan, we shall first direct our attention to the abnormal conditions of the teeth and gums during dentition. I have already described to you the symptoms which bring in and accompany the eruption of the milk teeth in the healthy subject: it now remains for me to direct your attention to the process when attended with unfavorable symptoms—to interrupted dentition.

The attendant symptoms are both local and general, but as the latter generally arise out of, and are consequent upon the local symptoms, we shall take the local condition of the gums as the base of this division of the subject.

*Interrupted dentition* is accompanied by either inflammation or induration of the gums, or by a combination of these conditions.

*Inflammation of the Gums.*—The local symptoms are: swelling, heat, redness, and pain, accompanied by excessive tenderness, so that the slightest pressure on the affected part becomes intolerable. These symptoms will vary in degree in different cases, and they will vary relatively also. In one case the swelling will be great, while the heat and redness will be moderate, and *vice versa:* aphthous ulceration not unfrequently accompanied with swelling and tenderness of the salivary glands. There is a profuse flow of viscid saliva. With these local symptoms, we have general disturbance of the system, evinced by a dry hot skin, rapid pulse, hurried respiration, thirst, broken sleep, vitiated and scanty secretions. But, in addition to these local and general symptoms of abnormal dentition, it not unfrequently happens that symptomatic affections arise, and these of no trivial nature. In the excited state of the system during painful dentition, inflammation either acute or chronic may attack any organ of the body. The organs of respiration, and the brain and nervous system, seem to be the most susceptible. Bowel complaints, terminating in mesenteric disease, sometimes supervene.

Many of the symptoms arising from interrupted dentition are of a purely nervous character. These vary, from slight spasmodic twitching of the muscles, to violent convulsions ending in death.

Sudden development of the teeth; the appearance of several teeth at the same time; the evolution of the teeth at an early age; unequal rate of development in the teeth and jaw, so that there is want of sufficient space for the teeth; delicate health of the patient; are enumerated as the causes of abnormal dentition, and the attendant diseases. But pressure produced by the growing tooth upon the inflamed gum, and upon the formative pulp, is considered as the more immediate cause of the active symptoms.

The inflammation of the gums, together with the symptomatic affections, not unfrequently precede some time the appearance of the tooth. The teeth are then said to be breeding. Towards the later stage of the process, the gums, instead of presenting uniform redness, are red only at the base, while that portion which lies over the coming tooth is tense, pale, and bloodless, a condition produced by the pressure of the tooth from within. In this and in the previous stages of the inflammation the epithelium peels off the surface of the affected gum, which bleeds from the slightest touch. If nature is allowed to take her course, the tooth after a while appears through the gum, and the little patient, if no serious coincident disease has established itself, returns to good health.

But instead of dentition proceeding, and nature working her own cure, serious maladies of fatal character may seize and carry off the patient in a few hours. Inflammation may attack a vital organ, and yield to no treatment. Cerebral symptoms may arise and terminate in coma or in fatal convulsions.

### *Abnormal States of the Teeth and Gums.*

| | | |
|---|---|---|
| Abnormal conditions during the presence of the milk teeth. | Diseases of the gums before the eruption of the teeth | Inflammation. Induration. |
| | Diseases of the dental tissues | Caries. Necrosis. |
| | Diseases of the gums after the eruption of the teeth | Inflammation. Ulceration. |
| | Diseases of the alveolar periosteum | Inflammation. |
| Abnormal conditions during the presence of the permanent teeth. | Irregularity in the time of eruption of the permanent teeth. | |
| | Irregularity in the position of the permanent teeth. | |
| | Mechanical injuries of the teeth | Fracture. Dislocation. |
| | Mechanical injuries of the alveoli | Fracture. |
| | Diseases of the dental tissues | Caries. Necrosis. Exostosis. Pain. |
| | Diseases of the pulp | Irritation. Inflammation. Ulceration. Granulation. |
| | Diseases of the alveolar periosteum | Inflammation. Suppuration. Hæmorrhage. |
| | Diseases of the alveoli | Inflammation. Necrosis. Absorption. |
| | Diseases of the gums | Inflammation. Recedence. Tumors. |
| | Tartar. | |

The primary or predisposing cause of the alarming diseases I have enumerated as connected with dentition, is often overlooked, from the very intensity of the diseases themselves, and attention is alone directed to their relief, without a thought being given to the predisposing cause; not that any local treatment addressed to the gums would remove inflammation affecting a distant organ arising as a consequence of irritation existing in the gums. Yet, if that irritation be removed, the chances of recovery will be greatly enhanced.

It will be your duty then, whenever called upon to treat a child suffering from disease, whatever be the nature of the disease (unless of specific character, such as small-pox,) to inquire into the state of dentition, and if you find the gums inflamed, and a tooth presenting, to freely lance the imposed gum. It is useless to lance the gum superficially; it should be divided down to the surface of the pressing tooth, even if this be at some little depth in the gum. Having relieved the state of the gum, attention must be directed to the coincident malady.

It has been supposed by some writers that lancing the gums before a tooth is ready to come through, retards rather than facilitates the future passage of the tooth. They consider that the cicatrix formed after the lancing is less readily absorbed than the gum would have been had there been no cicatrix, and, therefore, that an obstacle is created by the operations; unless, indeed, the tooth is so near the surface that it passes through the wound before the gum can heal. The assumption is not borne out by observation; cicatrices are more readily absorbed than natural tissues. You have many opportunities of observing how prone a cicatrix is to absorb, in the frequent occurrence of ulcers on cicatrices of the leg, and other parts of the body.

### *Induration of the Gums.*

Instead of the gum becoming removed for the passage of the teeth, we sometimes find it becomes indurated and thickened. In this condition there is no considerable tenderness, scarcely any increase in the quantity of saliva. The gum on inspection is elevated, but not more florid than the neighboring parts. Indeed, the local symptoms are slight, and often overlooked.

In connection with interrupted dentition, accompanied with these local conditions, the general symptoms are not less severe than where there is inflammation of the gums. The skin is hot and dry, the appetite fails: the child will often refuse the breast for an unusually long period. This state of health is often attributed to the presence of worms, and medicines are given to effect their expulsion.

In induration as in inflammation of the gums, either inflammatory or spasmodic diseases may arise and require active treatment, the remedies being such as the character of the disorder may indicate; but in no instance must we neglect to make free incisions in the gums over the teeth that are bound down by the hardened tissue.

Where the symptomatic disease is of a purely spasmodic character, this simple operation will sometimes produce instant remission of the symptoms, but when the disorder has advanced to alteration of structure, the most we can expect will be a remission in the intensity of the symptoms, and a state of system more favorable for the action of remedies useful in the treatment of the disease.

Dr. Ashburner has written a very valuable work on dentition, and its coincident disorders, in which a great number of cases are given, where lancing the gums produced instant relief to serious and distressing spasmodic diseases. The two following cases are taken from his work.

"I attended a fine boy from the cutting of its first incisor tooth to the completion of its dentition of twenty teeth. It was the last child of a family in which all the children had afforded examples of abnormal dentition. This boy was of a nervous temperament, with black hair and eyes: every tooth had come forward with a want of biliary secretion. Nothing could exceed the care observed by the mother as to the diet of this infant; yet, whenever an effort at developing a tooth took place, she was always aware, from the deficiency of bile in the evacuations, that he was to have slight fever, sometimes with a catarrh and cough, always with twitchings of the face and fingers, and starting and moaning during sleep, and a catch in fetching a deep sigh. With the appearance of the four first molares the spasms were more severe. The thumb of the right hand was thrust into the palm, and the fingers clenched upon it; the toes were drawn down, the face was distorted. These spasms relaxed and reappeared. I found on these occasions that the tooth was always abnormal in its progress; seldom observing its turn, and never its time. The gum lancet freely used always cured these spasms. On the last occasion I was sent for in great haste, for the spasms had continued into an epileptic fit, from which I speedily and effectually relieved my little patient, by cutting through the capsule of the coming tooth."

"I was called one evening to the child of a lady in Regent street. It was a fine girl of fourteen months old. The head was large; dark hair, dark eyes. It was in a tub of warm water, quite insensible, passing from a state of epilepsy into tetanus. Its eyes were not influenced by light; the pupils remained dilated. The anterior molaris of the left side, in the upper jaw, was ready to come through. I cut freely through its capsule with my gum lancet; in two minutes the child was on its knees in the water, playing with the handle of the tub, in excellent spirits. This child had been twitching the angle of its mouth, and had had its right arm spasmodically affected during the day."

Mr. Bell, in speaking of the diseases consequent upon the irritation produced by abnormal first dentition, and in reference to the frequent occurrence of diseases of the brain from this cause, makes the following remarks:

"The brain, too, is frequently affected; the pupils of the eyes are permanently expanded, the head is continually to and fro, with an uneasy restless motion, accompanied with an increased moaning. Spasm of the voluntary muscles frequently succeeds, and convulsions at length recur at intervals, with increasing violence; which, unless immediate relief be obtained, too often terminate at once the little patient's sufferings and existence. The symptoms which I have here described as connected with a morbid state of the brain, often arising either from the overcharged condition of the vessels, or from actual effusion, do not always terminate fatally; but even where they are for the time relieved, and their acute form subdued, the occurrence of confirmed hydrocephalus is amongst the most common results of such affections. It appears, indeed, that the irritation has not been sufficiently attended to as the origin of this disease. Were this view of its cause more generally taken, it is, I think, probable that it may, in many cases, be arrested in its earlier stages, and its dreadful consequences, death, or a state of idiocy far worse than death, be frequently prevented."

### *Caries and Necrosis of the Temporary Teeth.*

The temporary teeth are subject to the death and decomposition of a portion, constituting what is generally called caries, and to death of the whole tooth, constituting necrosis of the tooth. The former of these affections is generally common to the temporary and permanent teeth, and will be considered when we come to the diseases of the permanent teeth. But necrosis is more frequently found in the milk teeth, and moreover presents some peculiarities.

Dental necrosis in the young subject is usually consequent upon the destruction of the whole or part of the crown by caries. The pulp, from the exposure is destroyed; after which, all vitality is lost in the fangs. Supposing absorption of the roots to have commenced, the process is arrested, the remaining portion of the fangs becomes discolored, and the enclosing alveolus becomes absorbed. The gum then inflames and ulcerates, and the fangs of the teeth are exposed on the surface, from which, in a little time, they become detached. In the process, the tooth itself is not unfrequently borne up towards the surface, and is found lying across the gum, with the fangs directed towards the cheek, which is not uncommonly ulcerated by the rough points irritating the mucous membrane.

It does not always happen that the inflammation produced by the necrosed tooth is slight in degree, or limited in its effects; on the contrary, the neighboring teeth and alveoli may partake of the diseased action, and suffer necrosis; and further, you will find in the separated sequestrum the rudiments of the permanent.

*Treatment.*—The dead tooth or teeth should be removed and the inflammation combatted by general treatment. If the ulcer in the cheek present an unhealthy appearance, the surface should be touched once or twice with sulphate of copper. If the alveoli are necrosed, a time must be given for the dead bone to be separated from the living; but so soon as this is effected, the sequestrum should be removed. During the time the above bone remains in the mouth a wash should be used, composed of chloride of lime and water, as a means of rendering the dead bone less offensive, and keeping the parts clean.

*Diseases of the Gums during the Presence of the Temporary Teeth.*

At this period of life the gums are liable to be attacked with inflammation and other diseases common to childhood and after life. These require no special notice in this division of our subject, but will come under our notice when treating of the diseases of the gums generally.

The gums are, however, during childhood subject to a peculiar form of ulceration closely allied to that form of disease, which occurring in the adult in any other situation than the mouth, is called phagedena, and which when seated in the mouth, is by most writers called cancrum oris. The disease commences on the free edge of the gum with inflammation, which is marked by swelling, vascularity, and lividity of surface. Ulceration then commences on the edge of the gum, and quickly extends towards the alveolus, destroying in its course the texture involved. The disease, in addition to extending towards the alveoli, proceeds to the gums of the neighboring teeth, but in these, as in those first attacked, the free edges of the gums are the parts first destroyed. The most common seat of the disease at its commencement is the edge of the gum about the canine and first molar. The gums of the lower jaw seem more liable to the disease than those of the upper maxilla.

In the cases which have come under my observation, the ulceration has commenced on the external edge of the gums, but has not with one exception, passed to the gums between the teeth, and thence to the inner gums. This may, however, have arisen from the cases being treated early, or being in themselves less severe than those occurring to other practitioners. The surface of the cheek lying in contact with the ulcerated surface of the gums becomes involved in a similar disease, so that the surfaces of the two ulcers lie in contact. The parts immediately surrounding the ulceration are thickened, indurated, and of a deep red color. The diseased action, in addition to extending over the surface of the gum, also affects in an almost equal degree the periosteal connection between the root of the teeth and the alveolus. The teeth become loose, and seem in their loose state to cause, by irritation, continuance of the disease. The teeth, instead of becoming merely loose in severe cases, are necrosed, blacken, and produce by their presence, as foreign bodies, an increase of the inflammatory action. The surface of the ulcer is so peculiar in character as to be immediately recognised; it is of a pale yellow or straw color, with here and there a red point, slightly raised above the general surface, which bleeds on the slightest touch. The disease in its progress quickly destroys the gum, and leaves exposed the alveolus, which then becomes necrosed. The ulceration, if unchecked by treatment, in unfavorable cases, extends, destroying in its course the texture of the mouth, till some large vessel is laid open, or the patient sinks from general exhaustion.

The causes of this destructive form of ulceration are for the most part constitutional. It is only seen in children of strumous habit, who from some cause are in a low and debilitated condition, and is most commonly found in those who have resided in damp and badly ventilated situations, and have been badly fed. When I held the office of dentist to King's College Hospital, a number of cases of this disease presented themselves, almost all of which came from the immediate neighborhood of Clare Market. At this hospital I have seen but three or four cases during the past two years, and these were slight only.

The peculiar form of ulceration which I have described has occurred to fearful extent in the children of the Asylum of Philadelphia. A memoir was published, by Dr. Coates, in "The North American Medical and Surgical," for July, 1826, in which is an excellent account of a number of cases terminating fatally.

But in these cases two forms of disease are described under one name, *cancrum oris*. The one, similar to that I have described to you as commencing in ulceration, about the edges of the gums; another, in which some part of the cheeks or fauces is attacked with sphacelus, succeeded by ulceration. The latter disease does not affect the gums, though it seems from Dr. Coates' paper to supervene in some cases upon ulceration of the gums. This disease, affecting other parts of the mouth than the gums, does not properly belong to our subject, and though in its nature allied to the corroding ulceration attacking the gums, will, I think, be found to possess points of dissimilarity sufficient to establish it as a distinct disease. For this reason I have not adopted the name of cancrum oris to the phagadenic ulceration of the gums.

*Treatment.*—To successfully combat the ulceration, both local and constitutional treatment must be adopted. The state of the bowels must be first attended to; a mild aperient should be given in the form of grey powder—this medicine answering the double purpose of stimulating the liver, which is generally defective in its secretions, and expelling any accumulation of acrid matter from the bowels. Tonics and liberal diet should then be administered. The local treatment must be similar to that adopted in ulceration of specific character, ulceration which increases by the acridity of its own secretion; that is, the surface should be perfectly destroyed by some agent which, by its chemical action, will decompose both the secretion and the surface of the sore. Nitrate of silver, or potassa fusa, or either of the mineral acids, will effect the purpose, but nitrate of silver is perhaps the preferable agent. In applying the remedy the surface should be first wiped dry, and then with a stick of caustic the ulcerated part rubbed over, taking care that no point remains untouched. The immediate effect of the nitrate of silver will be marked by a change of color of the surface to which it has been applied.

In no case under my own observation has this treatment failed. The superficial slough, caused by the nitrate of silver, has in the course of two or three days separated, leaving a healthy ulcer, which has readily healed.

Supposing any teeth have been rendered loose by the disease they must be removed, otherwise they will keep up the action. The American author, in the cases to which I have alluded, used with success sulphate of copper as a local application.

The copper in my hands has not been equally successful. After using it I have been obliged to have recourse to the nitrate of silver. In a few obstinate cases, where the patient has been in wretched general health from want of proper or sufficient food, I have found it necessary to repeat the application of caustic twice or even three times, at intervals of six or eight days, before the ulcer assumed a healthy surface.

My friend Dr. West tells me that he has succeeded in curing the disease by the administration of chloride of potassa in small doses, repeated every four hours, and without having recourse to any local treatment.*

### *Disease of the Alveolar Periosteum of the Temporary Teeth.*

The alveolar periosteum, which connects the fang of the tooth to the enclosing alveolus, is subject to diseased action. It may become generally inflamed, or a certain portion only may be affected. The cause which most frequently produces disease in this situation is caries, or necrosis of the tooth; in which case the inflammation will at first be confined to the part surrounding the apex of the root, from which the membrane becomes separated. Pus is excreted from that surface, which is no longer adherent to the fang, so that the apex of the latter is placed in an abscess. Inflammation of the alveolar periosteum is recognised by pain in the tooth, extending to the jaw; slight elongation of the tooth, which gives pain when pressed upon; swelling, with heat and redness in the gum over the alveolus containing the tooth. If allowed to take its course the external swelling will increase, and the cheek will become more or less swollen. The skin hot and dry. A portion of the alveolus, opposite the external gum, will become absorbed, and allow the pus to advance towards the surface. In the course of a few days the swelling in the gum will become soft; the surface at

* This treatment was, I believe, first made generally known to the profession by Dr. Heat, in a paper published in the Medico-Chirurgical Transactions of last year.

some point will give way, and pus will escape. The symptoms will then subside, leaving a small fistulous opening, surrounded with two or three granulations communicating with the interior of the abscess; and the tooth will give no further trouble until, from some accidental cause, the orifice through which the discharge has taken place has closed, when we shall again have all the symptoms of alveolar inflammation, terminating in abscess.

*Treatment.*—The tooth which has induced the malady should be immediately extracted, and the disease will be cut short. If pus has formed, the gum should be freely lanced, in order to facilitate the escape, though if the tooth be extracted this will be unnecessary, unless we find it between the gum and the surface of the alveolus.

### *Abnormal Second Dentition—Irregularity in the time of the Eruption of the Permanent Teeth.*

Two separate actions are concerned in the renewal of the teeth; one action, the removal of the temporary teeth; the other, the evolution of the permanent.

For the regular appearance of the second teeth it is necessary that each of these should proceed uninterruptedly, and that the jaws should have attained the requisite size. These conditions, from causes which it is not always easy to estimate, are not in all cases fulfilled.

The fangs of the temporary teeth in some cases remain unabsorbed, and the teeth keep their position to the exclusion of those that should take their place. This cause of irregularity in time most frequently occurs in the milk molars than to either of the anterior teeth. During the last two years seven cases have come under my notice, in which, at the age of from 15 to 27 years, one or more of the bicuspides were absent, and their predecessors, the milk molares, present. The patients have come to the hospital from suffering pain in the temporary teeth. On removing the temporary, the succeeding permanent teeth were seen coming forward.

The reason for assuming that in these cases the permanent were kept back by the temporary teeth is, that the crown of the former is received between the fangs of the latter, and therefore cannot come through without the previous removal of the superimposed tooth. Again, irregularity in time seldom occurs to the same degree in any of the more anterior teeth, which, when the temporary teeth remain after the usual time for their ejection, appear either anterior or posterior to them.

Another cause of irregularity in the time of eruption of the adult teeth, arises from insufficient space in the jaw, from defective growth. The canine teeth are retarded from this cause more frequently than any other teeth, arising from the canine being last to be cut, and the space being occupied by the incisores and bicuspides which have been previously evolved. I have seen several specimens of this—one on the table—in which the canine tooth, from want of space, has been developed, but remained deep in the jaw, completely imbedded in bone, the crown resting between the fangs of the lateral incisor and the first bicuspides. Nature, in these cases, has fitted the teeth for their peculiar position by developing an extremely short fang.

The symptoms produced by interruption to the evolution of the permanent teeth, which are preceded by temporary teeth, are seldom severe; and, indeed, indications are not unfrequently wholly absent. When they are present, pain is felt about the obstructing temporary tooth, extending to the angle of the jaw. The pain is far from constant, coming on and going off at uncertain intervals. The gum round the tooth is generally slightly inflamed. The indications subside on the removal of the obstructing tooth.

The wisdom teeth are liable to great irregularity in the time of their appearance, which arises from want of space in the jaw, or from irregularity in the position in which the development has taken place. Varied and distressing symptoms arise from obstruction to the development of these teeth; but as these obstructions mostly arise from irregularity in the position of the teeth, we shall postpone entering into the subject until we come to the consideration of the irregularities in the position of the permanent teeth.

### LECTURE VIII.*

**IRREGULARITY IN THE POSITION OF THE PERMANENT TEETH—GENERAL REMARKS—ANTERIOR POSITION OF THE UNDER TEETH, OR "UNDERHUNG"—ANTAGONISM OF THE CUTTING EDGES OF THE UPPER AND LOWER FRONT TEETH—EXCESSIVE PROMINENCE OF THE UPPER FRONT TEETH.**

GENTLEMEN:—To-day I shall bring to your notice the various deviations from the normal arrangement of the teeth. You have been told that the front teeth, (by which I mean the incisores and canines,) with the bicuspides, occupy in the well-formed jaw a semi-circle, and that the molares extend backwards from the two ends of the semi-circle, in slightly diverging lines; thus forming together an elliptic curve. In this curve no tooth projects before or recedes behind the neighboring teeth. Such is the normal arrangement of the perfectly developed teeth in well formed jaws. Instead, however, of the teeth presenting this even and uninterrupted line, we occasionally find that one or more teeth are placed either external or internal to the line, the teeth themselves being at the same time individually well formed. In the normal position each tooth has its anterior and posterior, or to speak more correctly, its buccal and lingual surfaces, placed at right angles to the radius of the curve which

* The paper on "Cancrum Oris," by Dr. Henry Hunt, to which I referred in my last lecture, is continued in vol. xxvi of the Medico-Chirurgical Transactions. Dr. Hunt gives the following as the result of his experience:

"The quantity of salt (chlorate of potash) I have been in the habit of prescribing varies from twenty to sixty grains, according to the age of the child, in divided doses, in twenty-four hours, dissolved in water: the beneficial effect is often observed on the following day, almost always on the second; the disagreeable fœtor soon lessens, the sores put on a healthy reparative action, the dribbling of saliva diminishes, and if there is mere ulceration, it speedily heals, if there is an eschar, it soon separates, and the sore granulates kindly. It is sometimes advisable, indeed necessary, that the aperient should be occasionally repeated."

it contributes to form; but a tooth may be twisted on its axis, and have these surfaces in a line with the radius, and present its sides instead of the broad surfaces, anteriorly and posteriorly, and thus constitute a form of abnormal position. Mr. Saunders, in his lectures before referred to, speaks of a case in which a front tooth was twisted half way round, so that the proper lingual surface was anterior. In the normal arrangement the anterior teeth of the upper close over and in front of the corresponding teeth of the under jaw. In some cases, however, from malposition of the teeth, or from want of a proper ratio in the growth of the upper and lower jaw, the upper teeth close posterior to those of the lower jaw—a condition commonly denominated "underhung."

Instead of the under teeth closing anterior to the upper, we find in some instances that they close upon the cutting edges of the upper teeth; this constitutes another form of irregularity, though partaking of the same character as the preceding form.

A third form of abnormal position occurs where the front teeth do not come together at all, a space of variable width separating them when the mouth is closed.

From what has been stated you will observe that the subject, so far as the front teeth are concerned, divides itself into two heads; first, abnormal position of the whole of the front teeth; and secondly, abnormal position of part of the front teeth, or perfect and partial irregularity of the front teeth.

The causes of abnormal position of the teeth are almost exclusively of a mechanical nature, and may be divided into four classes. First, want of sufficient room in the alveolar arch for regular arrangement. Secondly, abnormal position during development, as regards the fangs of the preceding temporary teeth. Thus, supposing the cutting edge of an incisor at the time of formation be placed anterior to instead of (as is normal) posterior to the fang of the milk tooth, the permanent tooth will come down and occupy a position anterior to the proper place. Thirdly, non-absorption of the fangs of the milk teeth, and consequent prolonged presence of the milk tooth; thus obliging the permanent tooth to take an anterior or posterior position.

Fourthly, a more rapid growth in the one jaw than in the other, producing undue prominences of the teeth or abnormal development of the jaws. In a preparation on the table the teeth are well formed, and arranged normally, yet when the jaws are closed and the molares brought in contact, the front teeth do not meet by a quarter of an inch, arising from the peculiar form of the lower jaw.

In the foregoing classification I have enumerated to you the principal kinds of dental irregularity, with the causes producing them. In practice, however, you will not be able to classify from its cause each case of dental irregularity in the manner I have done in this description; on the contrary, you will meet with cases in which the producing cause partakes in some degree of the character of each division, and yet does not belong to either exclusively. However, by adopting this somewhat arbitrary division of the subject, I think I shall be able to give you directions for recognizing the cause of irregularity, and the treatment to be pursued; and in a more intelligible form than though we treated the matter with less system.

It is only necessary to recognize the cause of dental irregularity to pronounce with certainty whether the evil admits of remedy.

If malformation of the jaw be the cause there is but little hope of remedy; if the jaws are too small to admit of the natural arrangement of the teeth, but are otherwise well formed, then we may, by the judicious application of mechanical means, with which I shall presently make you acquainted, reduce the teeth to their normal position.

*Treatment.*—When we see how much can be done by orthopædic surgery in restoring crooked and deformed limbs to the natural form, whether the patient be middle-aged or young, we should at once conclude, even without the aid of experience, that much might be done to remedy irregularity in the arrangement of the teeth, and we should become more certain of our point when we observe that if the molar teeth from age or accident be lost, the under incisores closing against the posterior inclined surfaces of the upper incisores, slowly but surely force the latter outwards.

But experience proves that when dental irregularity arises from any other cause than malformation of the jaws, the malplaced tooth or teeth may, by steady and continuous pressure, be reduced to the proper position: sufficient alveolar space, of course, having been by some means gained.

The treatment, then, mainly consists in applying steady uniform pressure upon the irregular tooth in the direction of the place you wish the tooth to occupy.

What has been said of irregularity in the position of the front teeth applies generally to irregularity in the bicuspides, as also to the molares, though the latter are less frequently subject to irregularity in position, and when it does occur, the degree is very slight, amounting, except in rare cases, to nothing more than the one standing a little out, and the next a little in, thus forming a zigzag line. The wisdom tooth, from its peculiar situation in the jaw, is liable to several characteristic forms of irregularity leading to great inconvenience; we will, therefore, notice these after going through the forms of irregularity with the treatment in the more anterior teeth.

From this general outline we will proceed to consider our subject in detail.

### *Complete Irregularity in the Front Teeth.*

It can scarcely happen that we have irregularity in the teeth of one jaw without a corresponding irregularity in the teeth of the other jaw; the one following as a consequence of the other.

The most common abnormal position occurs in those cases where the upper teeth, on closing the mouth, pass behind those of the under jaw. This condition, when once established, is permanently maintained by the teeth of the under jaw, for where there is any force tending to press the upper teeth outwards into their proper position, the act of closing the mouth, and the consequent pressure of the under teeth upon the anterior surface of the upper teeth, would press them inwards, and thus overcome the force employed by nature to remedy the defect.

This form of malposition of the teeth may arise from several causes. Thus the under jaw and teeth may be developed more rapidly than the upper. The incisores of the under jaw usually appear before those of the upper jaw, and take their position internal to the temporary teeth, which at the time are usually loose and quickly fall out, and then the succeeding teeth come forward and occupy their places. But while this is going on, the upper incisores usually appear through the gum, coming through internal to the temporary teeth. Supposing, however, that the temporary are not loose, but retain their places, the permanent teeth are then forced still more internally, and if the front teeth of the under jaw have progressed in their development they will close between the temporary and permanent teeth of the upper jaw. The teeth having once assumed this position, necessarily retain it, unless mechanical means are resorted to for remedying the defect.

A third cause of the posterior position of the front teeth of the upper jaw may arise in the permanent teeth of the under jaw, coming through anteriorly to the temporary teeth, so that the upper teeth, closing behind them, tend to force them still further outwards.

*Treatment.*—The causes of the reversal of the relative position of the front teeth of the two jaws you have seen is purely mechanical, and that the defect is maintained by a mechanical cause. Our treatment must therefore be mechanical also. The first thing we have to do is to prevent the front teeth from closing, thus disabling them from exercising any influence upon each other. This is effected by placing caps of metal or ivory upon the masticating surface of the molares of the upper jaw, and of such a thickness that when the mouth is closed the front teeth no longer meet. Having attained this point, a steady uniform pressure must be directed against the posterior surface of the misplaced teeth, and continued till, on removing the caps upon the molares, the upper teeth are found to close in their natural position: the position having been once gained is retained by the action of the under teeth upon the inclined posterior surface.

There are three methods of applying the pressure to force the teeth outwards. In one a fixed point is gained by fitting a piece of ivory to the hard palate and the surfaces of the necks of the teeth, to which it is tied so as to prevent the possibility of motion: pieces of dry compressed wood are then interposed between the ivory and the necks of the teeth you wish to be moved outwards. The wood on absorbing moisture swells, and as the fitted ivory cannot be moved backwards the teeth are necessarily moved forwards. After the wood has remained about eight and forty hours, it should be removed and a fresh piece of dry wood put in its place, which on swelling, force the teeth still further outwards. The renewal of the wood is repeated till the teeth have assumed the required position; the ivory is then removed from the palate and the caps from the teeth, and the process is allowed to be completed by the action of the upper and under teeth upon each other: the under forcing the upper outwards, and the upper pressing the under inwards, till the molar teeth come in contact, when the front teeth assume their permanent place.

In the second method the fixed point is made anterior to the teeth, and is accomplished in the following manner: Caps, as in the former case, are fixed upon the molar teeth; from thence a strong piece of flattened gold or silver wire is made to pass in front of the teeth. Opposite each tooth the wire is perforated with one or two holes, through which a ligature of silk is passed round the neck of the tooth; the silk is then tied tightly to the wire. The teeth by the action of the silk are dragged outwards. The ligature requires to be tightened or removed from time to time till the teeth have assumed the required position, when the apparatus is removed. Instead of silk the vulcanised India-rubber is frequently used. The third method of treatment consists in fitting metal to the surface of the front teeth of the lower jaw. The plate is continued upwards above the edge of the teeth, but instead of proceeding in the same line, is turned inwards, so that on an attempt to close the mouth the piece of metal passes behind the teeth of the upper jaw, and presenting an inclined plane, forces them outwards. The success of the method of treatment will be greatly enhanced by capping the molar teeth.

Of the three forms of treatment I have described to you I much prefer the first, being more easy of application, more rapid in its effects, and less troublesome to the patient than the two succeeding methods.

A second form of complete irregularity of front teeth is when the upper and lower close upon each other. In this case the cutting edges are quickly worn down, and the teeth look short and stunted, or, losing the molares, the front teeth then become worn down to the level of the gums, and the pulp cavity filled by vascular dentine, rendering the remaining portion of the tooth solid.

This condition differs only in degree from that which I have described, and though, as far as personal appearance is concerned, it is less objectionable than that, yet in its effect it is more injurious. Where the upper teeth close behind the under, the teeth themselves are not injured, but where they close upon each other the thin edges are soon worn, and the teeth cease to be strictly incisores, but are capable only of crushing.

The treatment necessary for the reduction of this irregularity is precisely similar to that required in the treatment of the "under lining."

However, in these cases, if attention is directed to the subject early, the simple pressure hy the thumbs of the upper teeth outwards will, in many cases, with a little care and perseverance, place the teeth in their proper relative position; especially if frequent attempts be made to place the under teeth by drawing back the teeth of the under jaw behind those of the upper jaw.

As a farther means of restoration, the teeth may be closed upon some substance, a piece of wood, or the handle of a teaspoon, and the spoon pressed downwards: by the motion the upper teeth will be pressed outwards by that portion of the spoon within the mouth, while the under teeth acting as a fulcrum will be pressed inwards. This simple operation will, if frequently repeated, tend to reduce the teeth to their proper situation.

A third malposition of the front teeth occurs in an excessvie prominence of the upper set. The front teeth press into the lips, are constantly exposed, and when the mouth is closed rest upon the under lip. This form of irregularity is more or less connected with the defective development of the superior maxilla. The alveolar processes are developed at a considerable angle with the general surface of the face, and the edges embracing the necks of the teeth, are, therefore, very prominent. This prominence might arise from several causes. Thus the permanent teeth might come down in front instead of behind the temporary teeth and their alveoli, and supposing the milk teeth to be present a little beyond the usual period would at once tend to the deformity in question.

Again, if the incisores are long, and the molar teeth short, the under incisores on the mouth being closed, will, instead of resting upon the basal ridge of the upper teeth, press down the inclination of the ridge, and, finally get fairly behind the teeth, and with their edges against the surface of the gums. We often see this state produced when, from age or some other cause, the molares and bicuspides are lost.

*Treatment.*—The first thing to be done towards remedying this evil is to remove one or two bicuspid teeth, so that space may be gained for bringing the teeth inwards, which may then be effected by pressure inwards, the fixed point being either external to the teeth in the form of a band of metal, or internal to the teeth in the form of a piece of ivory fitted to the palate.

Before, however, we proceed to the remedy, we must look closely to the cause which has produced the evil or which keeps it up.

