# Correction

**Published:** 1848-01

**Authors:** 


*Correction.*—We published in the last number of the Journal, an extract of a letter from Dr. E. Townsend of Philadelphia, giving a description and drawing of a tongue-holder. This letter was addressed to Dr. Westcott, and only that portion of it which contained the description of the instrument was sent to us for publication. We have since learned by letter from Dr. Townsend, that he was not the inventor, but that the credit of this belongs to Mr. Lawrence, an ingenious young gentleman a student of his.—*Balt. Ed.*

